# Stochastic Dynamics of Fusion Low-to-High Confinement Mode (L-H) Transition: Correlation and Causal Analyses Using Information Geometry

**DOI:** 10.3390/e26010017

**Published:** 2023-12-22

**Authors:** Eun-Jin Kim, Abhiram Anand Thiruthummal

**Affiliations:** Centre for Fluids and Complex Systems, Coventry University, Coventry CV1 2TT, UK; thiruthuma@uni.coventry.ac.uk

**Keywords:** magnetic fusion plasmas, non-equilibrium statistics, L-H transition, bifurcation, information geometry, non-perturbative analysis, causality

## Abstract

We investigate the stochastic dynamics of the prey–predator model of the Low-to-High confinement mode (L-H) transition in magnetically confined fusion plasmas. By considering stochastic noise in the turbulence and zonal flows as well as constant and time-varying input power *Q*, we perform multiple stochastic simulations of over a million trajectories using GPU computing. Due to stochastic noise, some trajectories undergo the L-H transition while others do not, leading to a mixture of H-mode and dithering at a given time and/or input power. One of the consequences of this is that H-mode characteristics appear at a smaller input power $Q<Q_{c}$ (where $Q_{c}$ is the critical value for the L-H transition in the deterministic system) as a secondary peak of a probability density function (PDF) while dithering characteristics persists beyond the power threshold for $Q>Q_{c}$ as a second peak. The coexisting H-mode and dithering near $Q=Q_{c}$ leads to a prominent bimodal PDF with a gradual L-H transition rather than a sudden transition at $Q=Q_{c}$ and uncertainty in the input power. Also, a time-dependent input power leads to increased variability (dispersion) in stochastic trajectories and a more prominent bimodal PDF. We provide an interpretation of the results using information geometry to elucidate self-regulation between zonal flows, turbulence, and information causality rate to unravel causal relations involved in the L-H transition.

## 1. Introduction

Magnetically confined fusion of high-temperature plasmas aims to provide limitless environmentally friendly energy. The main challenge in achieving this aim is to maintain a high temperature of the core plasmas for a sufficiently long time to extract enough fusion energy that exceeds the energy input needed to heat the plasmas. This is the so-called confinement problem, which has been difficult to address, as plasmas in fusion devices tend to be unstable with various instabilities and become turbulent, with anomalous loss of energy, much larger than what is expected from collisional processes [1]. Furthermore, plasmas can be far from equilibrium with strong time-varying fluctuations where the traditional equilibrium statistical theory based on small fluctuations and short memory time become invalid. Examples would include avalanche-like events that can play an important role in turbulent transport [2,3,4,5], e.g., through their interaction with shear (zonal) flows.

One of the promising candidates for improved confinement regimes in fusion reactors is the high-confinement mode (H-mode), where the confinement time is increased by roughly a factor of two compared with the low-confinement mode (L-mode). The transition from the L-mode to H-mode, the so-called L-H transition, is considered to be a bifurcation of edge plasmas where the order parameter is a radial electric field E , which drives an E×B shear flow in the poloidal direction. Here, B is a magnetic field, which has a strong toroidal component in conventional tokamaks. The L-H transition has been one of the most active area of fusion research over the last 40 years since its first discovery in the 1980s [6] due to its reproducibility and importance for future fusion devices (such as ITER) [7] and STEP [8], among others.

More specifically, the L-H transition occurs as the input power approaches a critical value—the power threshold—and is believed to be caused by the turbulence reduction by radially sheared E×B flows [9,10,11,12,13,14,15,16,17,18]. The latter can involve two types of flows—mean shear flows driven by the mean pressure gradient and zonal flows internally driven by turbulence. As zonal flows grow from turbulence and then regulate turbulence when they are sufficiently strong, zonal flows and turbulence constitute a self-regulating system, their time-evolution leading to prey–predator type limit-cycle oscillations, commonly observed in population dynamics. Given the ubiquity of self-regulation and shear flow suppression of turbulence [19,20,21,22] in many other systems including astrophysical, geophysical, and environmental dynamics, the study of the L-H transition will help us understand other systems.

Of particular note is the universality of the L-H transition across models, fusion devices, and experiments. Specifically, the L-H transition has experimentally been observed in different tokamaks and reversed pinches and simulated using different approximations of plasma turbulence ranging from reduced ODE to fluid to gyrokinetic models [9,10,11,12,13,14,18]. The scaling relation of the power threshold is studied in terms of the mean values of several important variables (toroidal magnetic fields, electron density, etc.). In particular, the qualitative feature of the L-H transition was modelled through a deterministic prey–predator model [12] involving a prey (turbulence amplitude), a predator (zonal flow), and a super-predator (mean flow), where a zonal flow was shown to facilitate the transition by reducing turbulence prior to the transition before the mean shear flow locks the plasmas in H-mode.

However, the turbulence characteristics in L-mode are very variable; for instance, the RMS values of fluctuating electron density and turbulence velocity are highly time-varying. Furthermore, there is growing evidence for micro avalanches or transport events [5] occurring on time scales smaller than a typical L-H transition time of O(1) ms in edge plasmas from experiments and simulations, suggesting the importance of stochastic noise in the L-H transition. Furthermore, experimental studies have shown a large scatter in the power threshold (e.g., see [23]). To address this, [24,25,26] investigated the effects of stochastic noise on turbulence amplitude and zonal flows by extending [12] to a stochastic prey–predator L-H transition model and calculated time-dependent probability density functions (PDFs) by solving the Fokker–Planck (F-P) equation [27]. The results revealed that the L-H transition can involve strongly non-Gaussian PDFs with multiple peaks and intermittent zonal flows, which can play a key role in suppressing turbulence. We [28] applied the PDF methods to analyze time-series data of density fluctuations, perpendicular velocity, and, more recently, magnetic fluctuations, confirming some of the findings from the stochastic prey-predator models in [24,25].

The main aim of this paper is twofold. The first is to elucidate the mechanism underlying multimodal PDFs found from the F-P approach [24,25,26] by investigating how each stochastic trajectory evolves over time, undergoes the L-H transition, and affects a PDF shape. The second is to investigate how zonal flows and turbulence interact and are causally related prior to and during the L-H transition. To address these, in this paper, we perform multiple systematic stochastic simulations using GPU computing [29] and present a thorough statistical analysis utilizing information theory [30,31,32,33], in particular, information geometric theory [34,35,36,37,38], summarized in Section 2.

The remainder of the paper is organized as follows. Section 2 summarizes information geometric theory and proposes an instantaneous transfer entropy to quantify time-varying statistics. Section 3 presents the model and Section 4 provides the power threshold $Q_{c}$ for the deterministic system. Section 5 and Section 6 discuss the results from δ function initial conditions and Gaussian initial distributions, respectively. Section 7 compares the results from additive and multiplicative zonal noises. Conclusions are found in Section 8.

## 2. Information Geometry and Instantaneous Transfer Entropy

Information theory has become increasingly popular since it provides a fundamental entity with which one can describe complex systems as well as connecting seemingly different phenomena in terms of “information” (e.g., [33]). We focus on information geometric theory, which is a subset of a broader information theory. In simple terms, information geometry quantifies the distinguishability of two probability distributions with a metric and provides a way of understanding the difference between any two PDFs [34,35,36,37,38]. For time-dependent stochastic processes, the concept of information rate was proposed by comparing temporally adjacent PDFs (see review papers [37,38]) and by quantifying the rate at which a PDF changes, which signifies how fast a system changes its statistical states over time.

Furthermore, while there are causality analyses using information theory, such as mutual information [39], transfer entropy [39,40,41,42,43,44,45], and information flow [46,47], their formulation is based on entropy, which is a global measure of a PDF that is insensitive to the local arrangement of a PDF. Furthermore, entropy-based causality measures may not pick up a sudden change in mean values, as shown for information flow [48]. In comparison, the causal information rate based on information geometry is sensitive to the local arrangement of a PDF (e.g., a PDF shape) with the capability of capturing an abrupt event [48].

In this section, we summarize the definitions of information rate and causal information rate while proposing instantaneous transfer entropy to analyze the results in Section 6.

### 2.1. Information Rate

In order to quantify the temporal change in a PDF, we use a dimensionless quantity, the information length L , and its time derivative $\Gamma =\frac{dL}{dt}$ [37,38,49,50], which are defined by

(1) $$\begin{array}{ccc} L\left(t\right)=\int_{0}^{t}\Gamma \left(t_{1}\right)dt_{1}, & & \;\;\Gamma {\left(t\right)}^{2}=\frac{1}{\tau {\left(t\right)}^{2}}=\int dx\frac{1}{p(x,t)}{\left[\frac{\partial p(x,t)}{\partial t}\right]}^{2}.\; \end{array}$$

The unit of Γ in Equation (1) is ${\mathrm{time}}^{-1}$ ; τ represents a dynamic time unit for how quickly a PDF p(x,t) changes; L(t) measures the clock time in units of τ(t) and quantifies the total number of statistically different states that *x* passes through between time 0 and *t*, starting from some initial PDF p(x,0) . Consequently, L(t) represents the cumulative change in p(x,t) , taking into account the uncertainty due to a finite width of p(x,t) . We note that L(t) depends on $p(x,t^{\prime })$ for all $t^{\prime }\in \left[0,t\right]$ depending on the evolution of p(x,t) and is independent of the (time-independent) change in variables. We showed that L , as a path-dependent measure, is useful for understanding dynamics that have a long memory time and hysteresis involved in phase transitions [49] (e.g., the L-H transition), while Γ is useful for quantifying correlations [37,50] and forecasting abrupt events [51]. In particular, a strong correlation between two switching species was captured by the similar evolution of L(t) of these two species [50] despite the different time-evolutions of their PDFs.

For a system with two variables $x_{1},x_{2}$ , we define the information length and rate for the *i*^th^ variables ( i=1,2 ) as

(2) $$\begin{array}{ccc} & & L_{i}\left(t\right)=\int_{0}^{t}dt_{1}\;\Gamma_{i}\left(t_{1}\right) \end{array}$$

(3) $$\begin{array}{ccc} & & \Gamma_{i}^{2}=\int dx_{i}\frac{1}{p(x_{i},t)}{\left[\frac{\partial p(x_{i},t)}{\partial t}\right]}^{2}.\;\;\; \end{array}$$

Since $\Gamma_{i}$ and $L_{i}$ depend on the time history of $x_{i}$ , we can quantify the correlation or causality between $x_{i}$ and $x_{j}$ ( i≠j ) by comparing $\Gamma_{i}$ and $\Gamma_{j}$ ( i≠j ).

### 2.2. Causal Information Rate

Ref. [48] proposed the causal information rate $\Gamma_{i\to j}$ for i≠j ( i,j=1,2 ) from the variable $X_{i}$ to $X_{j}$ using a bivariant joint PDF $p(X_{i},t_{1};X_{j},t_{2})$ at different times $t_{1}$ and $t_{2}$ , its equal-time joint PDF $p\left(X_{i},t;X_{j},t\right)\equiv p\left(X_{i},X_{j},t\right)$ , the conditional entropy $p\left(X_{j},t_{2}|X_{i},t_{1}\right)=p\left(X_{j},t_{2};X_{i},t_{1}\right)/p\left(X_{i},t_{1}\right)$ , and the marginal PDFs $p\left(X_{i},t\right)=\int dX_{j}p\left(X_{i},X_{j},t\right)$ and $p\left(X_{j},t\right)=\int dx_{i}p\left(X_{i},X_{j},t\right)$ as follows:

(4) $$\begin{array}{ccc} \Gamma_{i\to j} & \equiv & \Gamma_{j}^{*}-\Gamma_{j}, \end{array}$$

(5) $$\begin{array}{ccc} {\Gamma^{2}}_{j} & \equiv & \Gamma_{j}{\left(t\right)}^{2}=\int dX_{j}\;p\left(X_{j},t\right){\left(\partial_{t}\ln(p\left(X_{j},t\right)\right)}^{2},\\ {\Gamma^{2}}_{j}^{*} & \equiv & \Gamma_{j}^{*}{\left(t\right)}^{2}=\lim_{t_{1}\to t^{+}}\int dX_{i}dX_{j}\;p\left(X_{j},t_{1};X_{i},t\right){\left(\partial_{t_{1}}\ln\left[p\left(X_{j},t_{1}|X_{i},t\right)\right]\right)}^{2} \end{array}$$

(6) $$\begin{array}{ccc} & = & \lim_{t_{1}\to t^{+}}\int dX_{i}dX_{j}\;p\left(X_{j},t_{1};X_{i},t\right){\left(\partial_{t_{1}}\ln\left[p\left(X_{j},t_{1};X_{i},t\right)\right]\right)}^{2}. \end{array}$$

Here, $\partial_{t_{1}}p\left(X_{i},t\right)=0$ for $t_{1}\neq t$ was used; $\Gamma_{i}=\frac{1}{\tau_{i}\left(t\right)}$ represents the information rate of $X_{i}$ with its characteristic timescale $\tau_{i}\left(t\right)$ ; $\Gamma_{j}^{*}$ represents the information rate of $X_{j}$ for a given (frozen) $X_{i}$ . Subtracting $\Gamma_{j}$ from $\Gamma_{j}^{*}$ in Equation (4) then gives us the contribution of dynamic (time-evolving) $X_{i}$ to $\Gamma_{j}$ , signifying how $X_{i}$ instantaneously influences the information rate of $X_{j}$ . That is, the causal information rate quantifies how one variable affects the change in the statistical state (PDF) of the other variable. Alternatively, it represents the effect of the dynamic change in the statistical state of one variable due to the other. $\Gamma_{i\to j}\neq \Gamma_{j\to i}$ in general, and the net causal information rate $\Gamma_{i\to j}-\Gamma_{j\to i}$ quantifies the net effect of *i* on *j*.

Here, note that Equation (6) can be shown to be related to the infinitesimal relative entropy (see Appendix A) as

(7) $$\begin{array}{ccc} {\Gamma^{2}}_{j}^{*} & = & 2\lim_{dt\to 0}\frac{1}{{\left(dt\right)}^{2}}\int dX_{i}dX_{j}\;p\left(X_{j},t+dt;X_{i},t\right)\ln\left[\frac{p(X_{j},t+dt;X_{i},t)}{p(X_{j},X_{i},t)}\right]. \end{array}$$

### 2.3. Instantaneous Transfer Entropy

One popular information theoretical method for quantifying causality is transfer entropy [39,40,41,43], which is based on the uncertainty reduction or the improvement in the prediction of one variable by having the knowledge of the behaviour of another variable at an earlier time. It was applied to understand causal relations involved in stationary data from fusion plasma [44,45]. However, the usual definition of transfer entropy given in [40] is only applicable to stationary states. Therefore, in this paper we define an instantaneous transfer entropy applicable for non-stationary systems:

(8) $$T_{i\to j}\left(t\right)=\lim_{dt\to 0}\int dX_{i}dX_{j}p\left(X_{j},t+dt;X_{j},t;X_{i},t\right)\log_{2}\frac{p\left(X_{j},t+dt;X_{j},t;X_{i},t\right)p\left(X_{j},t\right)}{p\left(X_{j},t;X_{i},t\right)p\left(X_{j},t+dt;X_{j},t\right)}.$$

Here, $T_{i\to j}$ denotes the instantaneous transfer entropy from $X_{i}$ to $X_{j}$ ; $p\left(X_{j},t+dt;X_{j},t;X_{i},t\right)$ denotes the joint PDF of $X_{j}\left(t+dt\right)$ , $X_{j}\left(t\right)$ , and $X_{i}\left(t\right)$ ; $p\left(X_{j},t+dt;X_{j},t\right)$ represents the joint PDF of $X_{j}\left(t+dt\right)$ and $X_{j}\left(t\right)$ ; and $p\left(X_{j},t\right)$ is the marginal PDF of $X_{j}\left(t\right)$ . We note that $T_{i\to J}$ is a directional quantity and is not symmetric in *i* and *j*. Furthermore, $T_{i\to j}$ and $T_{j\to i}$ can take either positive or negative signs or vanish; the net causality from *i* to *j* is quantified by $T_{i\to j}-T_{j\to i}$ (e.g., see [44]).

Note that in the original definition of transfer entropy (e.g., see [40,44,45]), the PDFs denote the distribution of values in a single time series trajectory and, hence, require stationarity to be meaningful, whereas in the case of instantaneous transfer entropy the PDFs denote the distribution of stochastic trajectories at a specific instance in time. Note also that for a Gaussian process, the transfer entropy is proportional to the Granger causality [42].

Furthermore, in analyzing time signals, the statistics needed to obtain joint/conditional PDFs are calculated by sampling over time with some time lags. In comparison, we can calculate the transfer entropy at any time from joint/conditional PDFs constructed from the stochastic trajectories and present the time-dependent transfer entropy.

### 2.4. Numerical Computation

Numerical computation of information geometric quantities and transfer entropy first involves the estimation of PDFs of the distributions of stochastic trajectories. This can be obtained by either solving the corresponding Fokker–Planck equation or by simulating an ensemble of stochastic trajectories and estimating the PDFs. In this work, we prefer the latter approach, since it scales better when large numbers of variables are involved and can handle δ -function initial conditions.

For estimating PDFs, 10 million trajectories were simulated using a GPU. The time steps were changed adaptively to meet an absolute local error tolerance of $10^{-6}$ . For generating plots of univariate PDFs, the number of bins was chosen to be $n=2.6N^{1/3}$ , where *N* is the number of samples, which is 10 million in this case. This is a modified colorblue version of Rice’s rule. The modification was made since the PDFs were often non-Gaussian with sharper peaks.

Computing the causal information rate using Equation (4) involves estimating a univariate PDF $p\left(X_{j},t\right)$ and a bivariate PDF $p\left(X_{j},t_{1};X_{i},t\right)$ . The estimates were made using histograms having 100 uniform bins along each dimension. The computation of instantaneous transfer entropy using Equation (8) involves univariate, bivariate, and trivariate PDFs. The requirement of estimation of a trivariate PDF limits the number of bins and 25 uniform bins were used along each dimension. Note that these PDF estimates can in principle be improved by using kernel density estimators, but the algorithm is computationally more expensive and requires a non-trivial choice of its bandwidth parameter for accurate estimates.

Equation (4) contains terms of the form $P\left(X,t\right){\left(\partial_{t}\ln P\left(X,t\right)\right)}^{2}$ . These terms are numerically unstable when P(X,t)→0 due to the presence of logarithms. Therefore, we use the following identity to perform the computation:

(9) $$P\left(X,t\right){\left(\partial_{t}\ln P\left(X,t\right)\right)}^{2}=4{\left(\partial_{t}\sqrt{P(X,t)}\right)}^{2}$$

The derivative can then be approximated using finite difference.

(10) $$\partial_{t}\sqrt{P(X,t)}=\frac{\sqrt{P(X,t+dt)}-\sqrt{P(X,t)}}{dt}+\frac{\delta [P]}{dt}+O\left(dt^{2}\right)$$

Here $O(dt^{2})$ is the truncation error in the finite difference scheme and δ[P] is the error in the term $\sqrt{P(X,t+dt)}-\sqrt{P(X,t)}$ . For the 1D PDF estimate using a histogram [52], $\delta \left[P\right]∼\sqrt{M/N}$ , where *M* is the number of bins and *N* is the number of samples. In this work, $M∼10^{3}$ and $N∼10^{6}$ and therefore, δ[P]∼0.03 . By minimizing the error term in Equation (10) with respect to dt , we obtain dt∼0.1 . Note that we neglected some factors that depend on P(X,t) and these will depend on the specific form of P(X,t) . A detailed numerical treatment of this problem can be found in [29]. Throughout the rest of this work we use the value dt=0.1 to approximate expressions in Equation (17) and Equation (8).

The numerical integrations were performed using the trapezoidal rule.

(11) $$\int_{a}^{b}f\left(x\right)dx\approx \frac{\Delta x}{2}\sum_{k=1}^{N}\left(f\left(x_{k-1}\right)+f\left(x_{k}\right)\right),\;\Delta x=\frac{b-a}{N}.$$

## 3. Stochastic Prey–Predator Model

We recall that the prey–predator L-H transition model [12] consists of three coupled ODEs for turbulence amplitude ϵ , zonal flow *v*, and density gradient *N* as follows:

(12) $$\begin{array}{ccc} \frac{d\epsilon }{dt} & = & N\epsilon -a_{1}\epsilon^{2}-a_{2}V^{2}\epsilon -a_{3}v^{2}\epsilon , \end{array}$$

(13) $$\begin{array}{ccc} \frac{dv}{dt} & = & \frac{b_{1}\epsilon v}{1+b_{2}V^{2}}-b_{3}v, \end{array}$$

(14) $$\begin{array}{ccc} \frac{dN}{dt} & = & -c_{1}\epsilon N-c_{2}N+Q. \end{array}$$

Here, $a_{i}$ , $b_{i}$ , and $c_{i}$ are non-negative constants, $V=dN^{2}$ (with *d* a positive constant) is the mean flow, and *Q* is the external heating that ultimately drives the entire system. Equations (12)–(14) are identical to Equations (6)–(8) in [12]; *v*, ϵ , and *N* here correspond to $V_{ZF}$ , E , and N , respectively, in [12].

In Equation (12), ϵ grows due to the linear instability of the density gradient and are damped due to nonlinear interaction and turbulence regulation by mean flows and zonal flows. In Equation (13), zonal flows grow from turbulence inhibited by the mean flow ( $1+b_{2}V^{2}$ ) and are damped due to linear (collisional) damping. The density gradient in Equation (14) relaxes by turbulent transport and a neo-classical/collisional effect while driven by the input power *Q*. We recall that the L-mode is a state of high turbulence ϵ and low zonal flow *v*; dithering is of moderate ϵ and *v*; H-mode is a quiescent H-mode with ϵ=v=0 in this model.

In this paper, we focus on the 2D stochastic version of Equations (12)–(14), as the previous studies [24,25]. Specifically, we first make the adiabatic approximation of *N* in Equation (14) as

(15) $$N=\frac{Q}{c_{1}x^{2}+c_{2}}.$$

We then rewrite Equations (12) and (13) in terms of $x=\pm \sqrt{\epsilon }$ and add the two stochastic noises ξ and η (which were not considered in [12]) as follows:

(16) $$\begin{array}{ccc} \frac{dx}{dt}=f+\xi , & & \;\;\;\;f=\frac{1}{2}\left[N-a_{1}x^{2}-a_{2}V^{2}-a_{3}v^{2}\right]x, \end{array}$$

(17) $$\begin{array}{ccc} \frac{dv}{dt}=g+\eta , & & \;\;\;\;g=\frac{b_{1}x^{2}v}{1+b_{2}V^{2}}-b_{3}v. \end{array}$$

Here, ξ and η are two independent δ -correlated Gaussian stochastic noises [27] that satisfy

(18) $$\begin{array}{ccc} \langle \xi \left(t\right)\xi \left(t^{\prime }\right)\rangle =2D_{x}\delta \left(t-t^{\prime }\right),\;\; & & \langle \eta \left(t\right)\eta \left(t^{\prime }\right)\rangle =2D_{v}\delta \left(t-t^{\prime }\right),\\ \langle \xi \left(t\right)\eta \left(t^{\prime }\right)\rangle =0,\;\;\;\; & & \langle \xi \rangle =\langle \eta \rangle =0, \end{array}$$

where the angular brackets denote averages. $D_{x}$ and $D_{v}$ are the amplitudes of the stochastic noises ξ and η , affecting *x* and *v*, respectively.

The parameter values in Equations (16) and (17) are chosen to be the same as those in [12,24,25], namely, $a_{1}=0.2$ , $a_{2}=a_{3}=0.7$ , $b_{1}=1.5$ , $b_{2}=b_{3}=1$ , $c_{1}=1$ , $c_{2}=0.5$ , and d=1 . The behaviour of Equations (16) and (17) together with the condition (15) very much depends on these parameter values, and our specific choice was made to qualitatively reproduce the L-mode, dithering, and H-mode as *Q* increases. For $c_{1}=1,c_{2}=0.5$ , Equation (15) sets the critical turbulence amplitude $x_{c}=\sqrt{\frac{c_{2}}{c_{1}}}=0.717$ above and below which the damping of *N* is dominated by the transport by turbulence $c_{1}\epsilon$ (typical of the L-mode) and the collisional damping $c_{2}$ , respectively. One of the consequences of this is discussed below in Section 4 and Section 5. Furthermore, the zonal flow generation is severely inhibited by the mean shear when $V>b_{2}^{-1/2}=1$ .

We note that [24] considered a linearly increasing input power Q(t)=0.03t+0.1 for t∈[0,50] ( Q∈[0.1,1.6] ), while [25] studied the forward and backward transitions associated with the L-H and H-L transition using a linear increasing and then decreasing input power. In both studies, the initial condition was a narrow Gaussian PDF $p\left(x,v,0\right)\propto {\exp[-((|x|-0.5)}^{2}+v^{2})/5\cdot 10^{-3}]$ centered around x=0.5 and v=0 .

## 4. Power Threshold $Q_{c}$ for the 2D Deterministic System

Before presenting the results for the stochastic system, it is useful to note that, for the deterministic system with $D_{x}=D_{v}=0$ in Equations (17) and (18) and a constant power *Q*, whether the system evolves to the H-mode or not in the long time limit depends on the initial condition x(0) and v(0) . For instance,

- For x(0)=0.5,v(0)=0.1,0.01 , $Q_{c}=0.832$ .
- For x(0)=0.707,v(0)=0.01 , $Q_{c}=0.919$ .
- For x(0)=0.5,v(0)=0.2 , $Q_{c}=0.830$ .

Here, $Q_{c}$ again denotes the power threshold in the deterministic model in Equations (12)–(14) above and calculated to the third decimal point.

**Figure 1:**
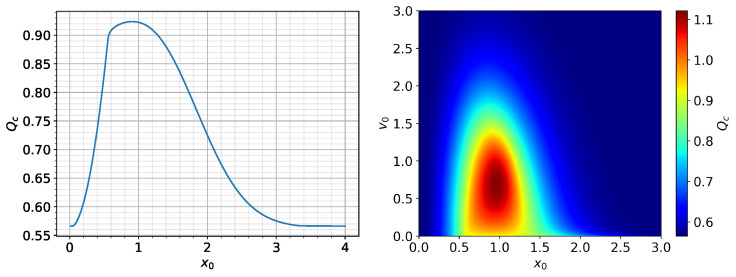
$Q_{c}$ vs $x_{0}$ with $v_{0}=0.01$ (**left**); $Q_{c}$ vs. $x_{0}$ for different $v_{0}=\left[0.01,3\right]$ (**right**).

In order to explore this dependence further, we first simulate 4000 different initial $x_{0}=\left[0.01,4\right]$ for a fixed $v_{0}=0.01$ and determine the corresponding $Q_{c}$ s. $Q_{c}$ is then plotted against $x_{0}$ in the left panel of Figure 1, which reveals three distinct regions. First, for small $x_{0}<x_{c}\;\left(\approx 0.7\right)$ , $Q_{c}$ increases with $x_{0}$ , suggesting that a smaller $Q_{c}$ is required when *Q* is applied suddenly (e.g., by a neutral beam) when the turbulence is rather weak.

Moreover, this region has a steady dithering solution (with an almost constant amplitude) for *Q* close to $Q_{c}$ ( $Q<Q_{c}$ ). Interestingly, recalling that collisional damping is more important than turbulent transport (as noted above) for $x_{0}<x_{c}$ , our result of a low $Q_{c}$ for a small $x_{0}$ is reminiscent of the observation that once plasmas are in the H-mode, the required power to keep the same plasma conditions is reduced due to increased energy confinement time (hysteresis effect) [23].

For the intermediate $x_{0}\approx \left[x_{c},2x_{c}\right]\approx \left[0.7,1.4\right]$ , $Q_{c}$ vs. $x_{0}$ shows a more or less parabolic behaviour and is associated with persistent finite-amplitude oscillations of the dithering before the transition to the H-mode. This is caused by the damping of *N* via turbulence transport, which is stronger than the collisional value for $x=x_{c}$ (as expected for the L-mode). On the other hand, too large an initial value $x_{0}>2x_{c}$ causes a rapid growth of zonal flows, which in turn damps turbulence quickly, essentially creating a scenario very similar to what happens to a small $x_{0}$ . This results in a decrease in $Q_{c}$ with $x_{0}$ .

In order to check the robustness of this tendency, we also explore the dependence of $Q_{c}$ on $v_{0}$ in addition to $x_{0}$ and present the results as a heat map in the right panel of Figure 1 using the color scheme shown on the right. The largest $Q_{c}$ (marked in dark red color) occurs for $x_{0}\approx 0.9,v_{0}\approx 0.7$ . Since these $x_{0},v_{0}$ values are the characteristic amplitude of a dithering solution, applying a constant *Q* in a dithering state will require a higher $Q_{c}$ to lead to the transition to the H-mode. Further discussion of this is provided in Section 8.

The heat map in Figure 1 around this maximum $Q_{c}$ exhibits a remarkable symmetry in $x_{0}$ and $v_{0}$ , with an approximately circular or triangular shape. This can be explained by the self-regulation between turbulence and zonal flow. In particular, for $x_{0}⪆0.9,v_{0}⪆0.7$ , $Q_{c}$ tends to monotonically decrease with $v_{0}$ for a fixed $x_{0}$ (or with $x_{0}$ for a fixed $x_{0}$ ), since a strong zonal flow $v_{0}$ rapidly damps turbulence, leading to a lower $Q_{c}$ . One conclusion that we could thus draw from these observations is that any physical mechanism that helps damping turbulence (e.g., a smaller collisional zonal damping) will facilitate the L-H transition with a lower $Q_{c}$ . Also, in a very weak turbulence regime $x_{0}\ll x_{c}$ , a sudden jump in *Q* would give a smaller $Q_{c}$ , as noted earlier. Physically, this would suggest that applying a set of neutral beams of a fixed voltage at the same time would be more advantageous than applying each beam individually at different times.

We will see shortly that a similar dependence of $Q_{c}$ on $x_{0}$ persists in the stochastic 2D model and discuss the implications.

## 5. δ -Function Initial Conditions and Constant Q

Here, we focus on investigating the effect of stochastic noise on $Q_{c}$ in comparison with the deterministic case. To this end, we use initial conditions that are given by a δ -function distribution. In passing, we note that in the previous study using the F-P method [24,25,26], δ -function initial conditions could not be implemented due to a resolution problem in the finite space difference numerical scheme. In comparison, a δ -function PDF is easily handled in stochastic simulations. This also allows us to explore the transition to the H-mode, where PDFs become very narrow, as shown below.

We first consider a δ -function initial condition of x(t=0)=0.5,v(t=0)=0.1 , in which case the power threshold is $Q_{c}=0.832$ in the deterministic model, as noted above. The strength of the stochastic noise is chosen as $D_{x}=D_{v}=10^{-4}$ . We explore how the system evolves to a statistically stationary state in the long time limit for a given constant *Q*.

**Figure 2:**
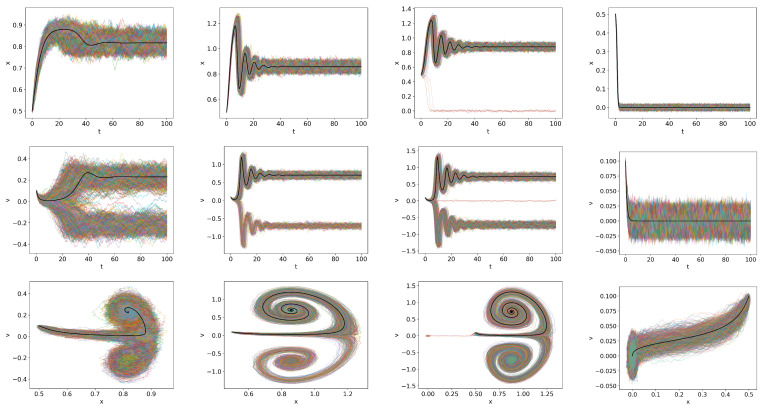
Trajectories of *x* and *v* and the phase portrait of x−v , respectively, in the first, second and third rows for δ -function initial condition with x(0)=0.5 and v(0)=0.1 , $D_{x}=D_{v}=10^{-4}$ ( $Q_{c}=0832$ ); Q=0.2,0.7,0.8,0.9 from left to right. The deterministic solution is over plotted by a thick black curve for comparison.

The main effect of the stochastic noises $D_{x}$ and $D_{v}$ is to induce stochastic trajectories, as can be seen in Figure 2, which plots the time-evolution of stochastic trajectories of 200 samples for Q=0.2,0.7,0.8,0.9 . The deterministic solution is over-plotted using a thick black curve. Due to the finite $D_{x}$ and $D_{v}$ , the same initial condition evolves to different stochastic trajectories. The larger $D_{x}$ and $D_{v}$ , the larger the dispersion (variability) in the stochastic trajectories. Also, due to the stochastic noise, some of the trajectories cross v=0 and take negative values v<0 .

To examine Figure 2 in detail, we recall that H-mode is characterized by x=v=0 , while dithering is characterized by finite values of x,v . For Q=0.2 , all the trajectories converge to a dithering state in the long term. For Q=0.7 , all trajectories again converge to a dithering sate. However, for $Q=0.8<Q_{c}$ , some of the trajectories converge to H-mode, while others remain in a dithering state, leading to a multimodal PDF. As *Q* increases, the peak at x=v=0 for the H-mode grows, while the one for dithering at finite x,v becomes smaller. When *Q* is increased to Q=0.9 , all the trajectories converge to the H-mode. Thus, the complete transition to the H-mode occurs at a slightly higher $Q>Q_{c}$ compared with the deterministic model, while the appearance of the H-mode characteristic appears for a smaller $Q<Q_{c}$ .

**Figure 3:**
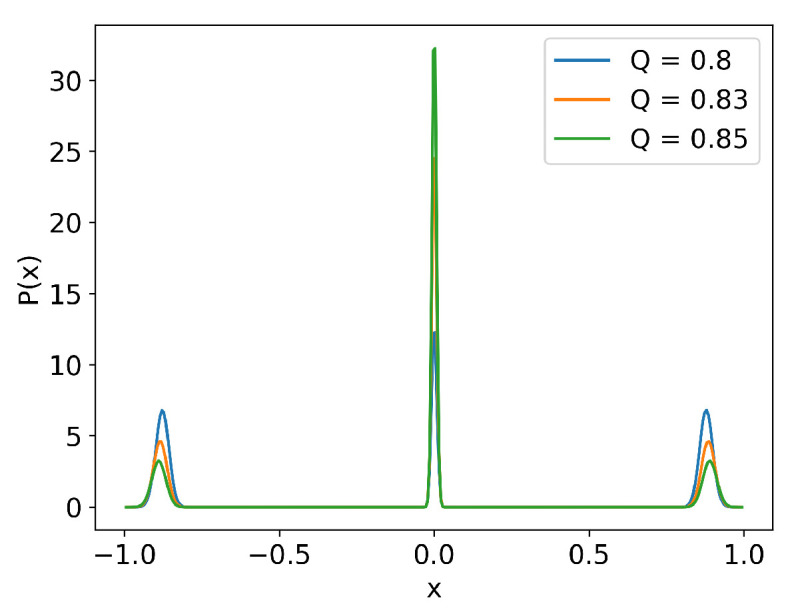
Stationary PDFs for Q=0.8,0.83,0.85 : δ -function initial condition with x(0)=0.5 and v(0)=0.1 , $D_{x}=D_{v}=10^{-4}$ .

**Figure A1:**
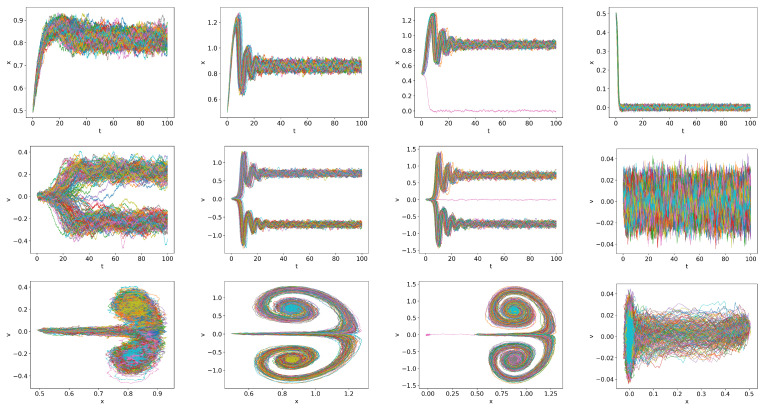
Trajectories for δ -function initial condition with x(0)=0.5 and v(0)=0.01 , $D_{x}=D_{v}=10^{-4}$ ( $Q_{c}=0832$ ); Q=0.2,0.7,0.8,0.9 from left to right.

To highlight this tendency, we show in Figure 3 the stationary PDFs of x,v obtained in the long time limit of the simulations of 2 million trajectories for constant power Q=0.80,0.83,0.85 . Quite similar behavior is observed for a slightly different initial condition x(t=0)=0.5 and v(t=0)=0.01 , and sample trajectories are shown in Figure A1 in Appendix B.

**Figure 4:**
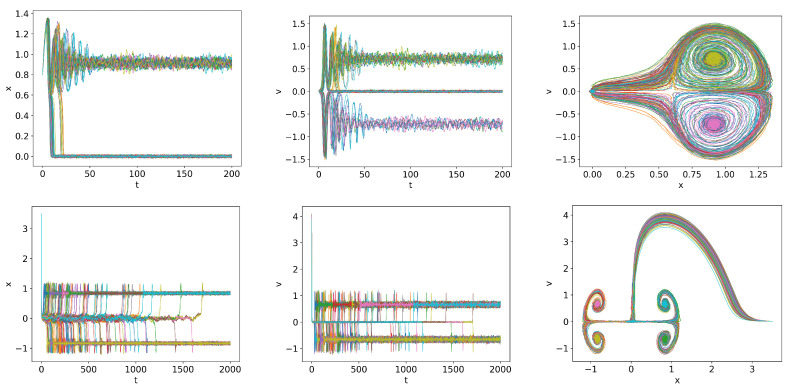
Trajectories for x(0)=0.8 and v(0)=0.01 at Q=0.95 and x(0)=3.5 and v(0)=0.01 at Q=0.58 in the top and bottom panels.

However, some initial conditions lead to quite different, interesting behaviour. Two examples that we show in Figure 4 are the trajectories for x(0)=0.8 and v(0)=0.01 at Q=1 and x(0)=3.5 and v(0)=0.01 at Q=0.58 . Robust oscillations during dithering are observed near $Q<Q_{c}$ in the upper panel. These dithering oscillations are very different from the bursts in the lower panel due to the intermittent switching between the H- and L-modes. Physically, the latter is due to the co-existence of two competing attractors and the stochastic switching between the two. This suggests the possibility of *alternation between L- and H-modes* just before the transition to the H-mode.

### Fraction of H-Mode and Comparison of $Q_{c}$ with the Deterministic Model

**Figure 5:**
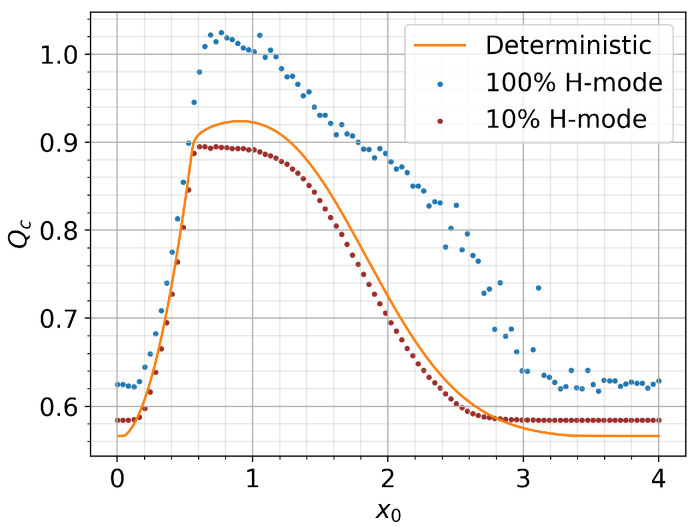
$Q_{c}$ vs. $x_{0}$ with $v_{0}=0.01$ .

In the previous section, we observed that in a stochastic model, a slightly larger value of *Q* is required for all the trajectories to converge to the H-mode in Figure 2. For convenience, we call $Q=Q_{100}$ the smallest *Q* where all the trajectories converge to the H-mode and present their values in blue dots in Figure 5. The deterministic $Q_{c}$ is also shown in the orange curve to help comparison.

However, we also observed that the H-mode characteristic appears for a lower $Q<Q_{c}$ , so we define $Q_{10}$ when 10 % of the population converge to the H-mode and show the results in the red dots in Figure 5. Obviously, $Q_{10}<Q_{c}<Q_{100}$ for moderate $x_{0}$ values between $x_{0}=0.2$ and $x_{0}=2.8$ , illustrating that the transition to the H-mode would occur rather gradually with *Q*. This physically means that there is uncertainty in the power threshold due to stochastic noise.

Furthermore, the overall dependence of $Q_{100}$ and $Q_{10}$ on $x_{0}$ is similar to $Q_{c}$ . Interestingly, this dependence of $Q_{100},Q_{10}$ on $x_{0}$ , in particular, a smaller $Q_{c}$ for a smaller $x_{0}$ ( $<x_{c}$ ), is consistent with the results of the recent paper [26], where one (big) single jump in *Q* to $Q_{*}>Q_{c}$ was found to facilitate the emergence of H-mode characteristics compared with the case of three (small) jumps to the same $Q_{*}>Q_{c}$ . Furthermore, these results highlight that time-scheduling of the heating could be an important factor contributing to $Q_{c}$ .

Note that in Figure 5, the $Q_{100}$ plot is noisy, since for some initial values $x_{0}$ and $v_{0}$ , H-mode is metastable with a long lifetime, as shown in Figure 4 bottom panel. The $Q_{100}$ values were computed using the bisection method, where the fraction of H-mode trajectories was calculated at each iteration by simulating 5000 trajectories for 2000 time units. The metastability combined with the finite duration of the simulation thus resulted in some error in the estimates.

## 6. Initial Distributions, Information Geometry, and Instantaneous Transfer Entropy

We now consider an initial condition that is given by a Gaussian distribution $p\left(x,v,0\right)\propto {\exp[-((|x|-0.5)}^{2}+v^{2})/5\cdot 10^{-3}]$ and $D_{x}=D_{v}=10^{-4}$ and use the same parameter values as before. We consider a constant *Q* and time-varying *Q* and provide a detailed statistical analysis using the information rate, causal information rate, and instantaneous transfer entropy defined in Section 2. The main aim of this section is to present the effects of stochastic noise due to the uncertainty in the initial conditions as well as provide a new statistical analysis of the L-H transition and its backward H-L transition using information geometric methods, which can help us better understanding of dynamic interaction and causal relations that are involved.

### 6.1. Constant *Q*

**Figure 6:**
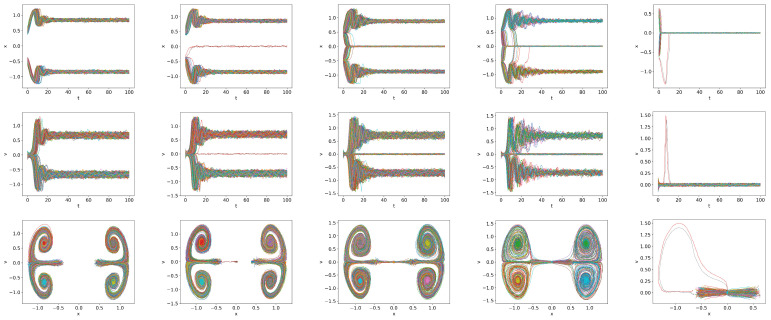
Initial condition $p\left(x,v,0\right)\propto {\exp[-((|x|-0.5)}^{2}+v^{2})/5\cdot 10^{-3}]$ and $D_{x}=D_{v}=10^{-4}$ . $Q_{c}=0.832$ . Q=0.6,0.7,0.8,0.9,1 from left to right, respectively.

The additive stochastic noise quickly randomizes the trajectories starting from the initial distribution, as can be seen in Figure 6 for constant Q=0.6,0.7,0.8,0.9,1.0 . Specifically, the H-mode characteristic starts emerging at Q=0.7 , smaller than Q=0.8 for the case of the δ -function initial condition in Figure 2. On the other hand, the H-mode characteristic persists up to a higher Q=0.9 in comparison with the δ -function initial condition. These results corroborate our previous observation that stochastic noise induces a more gradual transition. Another distinct difference is the almost symmetric *x* trajectories due to the symmetric initial distribution. The appearance of some of the non-*x*-symmetric trajectories in *Q* = 0.7, 0.8, 0.9, 1 is due to the finite number of simulations. That is, the trajectories will become symmetric in both *x* and *v* for a sufficiently large number of simulations.

### 6.2. Time Varying Q = 0.01t , 0.03t , 0.1t

**Figure 7:**
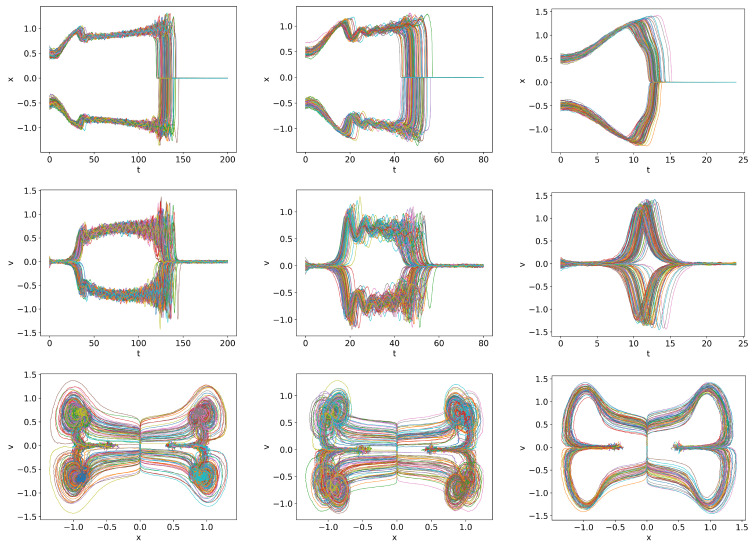
Initial condition $p\left(x,v,0\right)\propto {\exp[-((|x|-0.5)}^{2}+v^{2})/5\cdot 10^{-3}]$ and $D_{x}=D_{v}=10^{-4}$ . $Q_{c}=0.832$ . Q=0.01t , Q=0.03t and Q=0.1t in first, second and third columns respectively.

We consider the cases where *Q* is linearly increasing far beyond $Q_{c}$ at the three different rates Q=0.01t,0.03t,0.1t . Note that stochastic simulations allow us to investigate the transition to the H-mode in the regime where PDFs become very narrow. Figure 7 shows the trajectories for *Q* = 0.01t , 0.03t , 0.1t from left to right. In all cases, a finite time interval over which the transition to the H-mode occurs is visible. Specifically, from left to right, these time intervals are approximately t=(110,150),(40,60),(13,18) , corresponding to Q=(11,15),(12,18),(13,18) , respectively, showing a tendency of a slight increase in *Q* for a rapid damping. This is due to the time lag between *Q* and the system’s behaviour, with a larger lag for a faster change in *Q*.

Of additional interest is that the variability in trajectories is most pronounced for Q=0.03t , in which case *x* initially rapidly increases to the value of x=1 , followed by prominent oscillations. This is consistent with our previous observation that the initial *x* conditions around x≈1 (the plateau region in Figure 1) have a long duration of oscillations before transitioning to the H-mode. For Q=0.1t , the *x* value increases to a larger value x>1 and then monotonically approaches the H-mode due to too fast a ramping.

**Figure 8:**
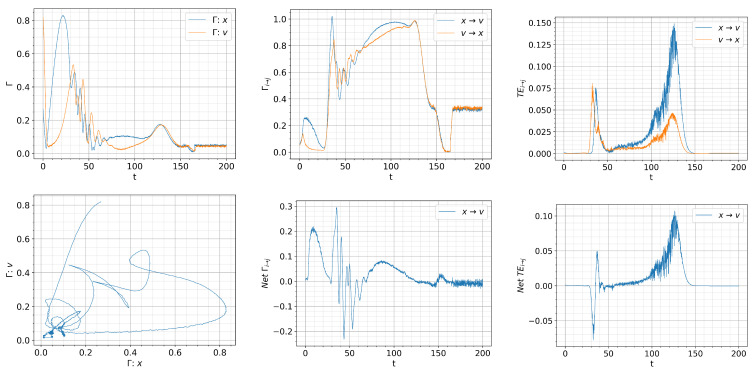
Initial condition $p\left(x,v,0\right)\propto {\exp[-((|x|-0.5)}^{2}+v^{2})/5\cdot 10^{-3}]$ and $D_{x}=D_{v}=10^{-4}$ . $Q_{c}=0.832$ . Q=0.01t . Top: from left to right, information rate, causal information rate and transfer entropy; bottom: from left to right, phase portrait, net causal information rate and net transfer entropy.

**Figure 9:**
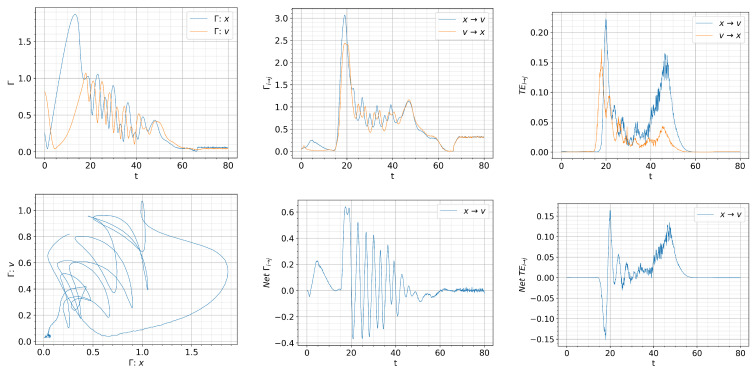
Initial condition $p\left(x,v,0\right)\propto {\exp[-((|x|-0.5)}^{2}+v^{2})/5\cdot 10^{-3}]$ and $D_{x}=D_{v}=10^{-4}$ . $Q_{c}=0.832$ . Q=0.03t . Top: from left to right, information rate, causal information rate and transfer entropy; bottom: from left to right, phase portrait, net causal information rate and net transfer entropy.

**Figure 10:**
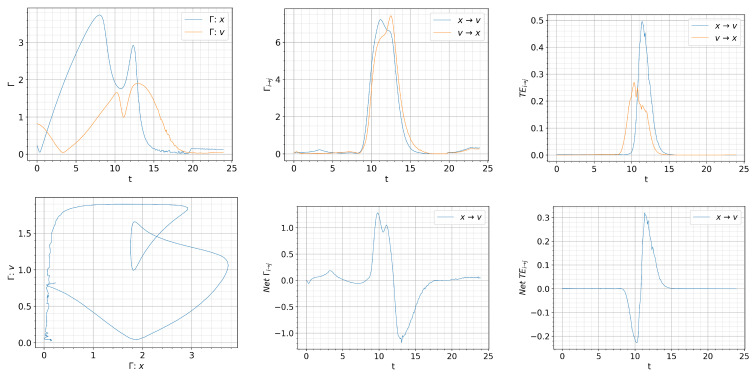
Initial condition $p\left(x,v,0\right)\propto {\exp[-((|x|-0.5)}^{2}+v^{2})/5\cdot 10^{-3}]$ and $D_{x}=D_{v}=10^{-4}$ . $Q_{c}=0.832$ . Q=0.1t . Top: from left to right, information rate, causal information rate and transfer entropy; bottom: from left to right, phase portrait, net causal information rate and net transfer entropy.

Figure 8, Figure 9 and Figure 10 show the information rate, causal information rate, and transfer entropy for Q=0.01t , Q=0.03t , and Q=0.1t . In each figure, the top panels show the information rate $\Gamma_{x}$ , $\Gamma_{v}$ , causal information rate $\Gamma_{x\to v}$ , $\Gamma_{v\to x}$ , and transfer entropy $T_{x\to v}$ and $T_{v\to x}$ from left to right columns; the bottom panels show the information phase portrait $\Gamma_{x}$ against $\Gamma_{v}$ , net causal information rate, and net transfer entropy. Here, net $\Gamma_{x\to v}$ is defined as $\Gamma_{x\to v}-\Gamma_{v\to x}$ , and similarly, net $T_{x\to v}$ is defined as $T_{x\to v}-T_{v\to x}$ .

For Q=0.01t , we observe a large spike in $\Gamma_{v}$ that appears at t=0 in the first column of Figure 8 due to a rapid evolution of p(v,t) towards an almost δ -function PDF around v=0 (L-mode). Self-regulation of x,v and the resulting dithering is manifested by the oscillations in $\Gamma_{x},\Gamma_{v}$ with approximately 180-degree phase difference for t≈(30,60) . The crossing of $\Gamma_{x}$ and $\Gamma_{v}$ signifies the time matching of the dynamics of *x* and *v* and can be clearly seen in the $\Gamma_{x}$ vs., $\Gamma_{v}$ phase portrait, where the trajectories are scattered around the diagonal line with unit slope around $\Gamma_{x}=\Gamma_{v}$ .

A very interesting feature can be inferred from the causal information rate in second column of Figure 8. In particular, for small times t=[0,30) in the L-mode, we observe $\Gamma_{x\to v}>\Gamma_{v\to x}$ , which suggests that the dynamics of turbulence (*x*) is mainly causing that of zonal flow (*v*). This is consistent with the expectation of *L*-mode dynamics. A large spike in the net causal information rate $\Gamma_{x\to v}-\Gamma_{v\to x}>0$ around t=30 captures a driving of zonal flow from turbulence, which rapidly increases the amplitude of *v* (as seen in Figure 7). This is followed by $\Gamma_{x\to v}-\Gamma_{v\to x}<0$ as *v* starts regulating *x*, and then the self-regulation of x,v where x,v are mutually influencing each other with the alternative sign in the net causal information rate $\Gamma_{x\to v}-\Gamma_{v\to x}$ .

In comparison, the instantaneous transfer entropy in the third column of Figure 8 does not seem to reflect the expected causal relations between *x* and *v* in the L-mode, L-H, transition and H-mode. In particular, for 30⪅t⪅35 , the net transfer entropy $T_{x\to v}-T_{v\to x}<0$ , implying that zonal flow is causing turbulence instead of the turbulence causing zonal flow (growth). This is followed by the net transfer entropy $T_{x\to v}-T_{v\to x}>0$ at 35⪅t⪅40 , occurring later than zonal flow growth which starts at t<30 . For 50⪅t⪅140 , the net transfer entropy $T_{x\to v}-T_{v\to x}>0$ , suggesting a stronger coupling from turbulence to zonal flow during the dithering and the transition to the H-mode, with no clear signature of what is causing the H-mode transition. This is to be contrasted to the net causal information rate $\Gamma_{x\to v}-\Gamma_{v\to x}<0$ around t≈130 , noted above.

We observed in Figure 7, dithering is most pronounced for Q=0.03t . Similar tendency can be seen in all panels in Figure 9. In particular, the time matching of $\Gamma_{x}=\Gamma_{v}$ is very well noticeable in the intervals $\Gamma_{x},\Gamma_{v}=\left(0,1\right)$ . And a similar conclusion can be drawn in regards to the causal information rate and transfer entropy.

For the fastest ramping in Figure 10, the change in the input power is too fast for the system to catch up, causing the system to be further from equilibrium. Thus, the overall values of $\Gamma_{x},\Gamma_{v}$ , $\Gamma_{x\to v},\Gamma_{v\to x}$ , and $T_{x\to v},T_{v\to x}$ are much higher than those for the slower ramping Q=0.01t,0.03t . Consequently, dithering and self-regulation are much less notable. Nevertheless, the regulation between x,v is well captured by $\Gamma_{x\to v},\Gamma_{v\to x}$ with their overall similar time-evolution.

### 6.3. Mirror-Symmetric Q = 0.01t , 0.03t , 0.1t

As in the previous work, we model the forward and backward processes associated with the L-H and H-L transitions using a mirror-symmetric *Q* at $t=t_{s}$ when *Q* takes its maximum value. For $t<t_{s}$ , *Q* linearly increases, while for $t>t_{s}$ , it linearly decreases. Specifically, we use the same three ramping rates as above apart from the mirror-symmetric *Q* around $t=t_{s}$ as $Q=0.01t,t_{s}=120$ , $Q=0.03t,t_{s}=40$ , and $Q=0.1t,t_{s}=12$ so that all three cases have $Q_{max}=1.2>Q_{c}$ .

The results equivalent to those in Figure 7, Figure 8, Figure 9 and Figure 10 are shown in Figure 11, Figure 12, Figure 13 and Figure 14, respectively. As they should be, the results in Figure 7, Figure 8, Figure 9, Figure 10, Figure 11, Figure 12, Figure 13 and Figure 14 are identical up to $t=t_{s}$ .

**Figure 11:**
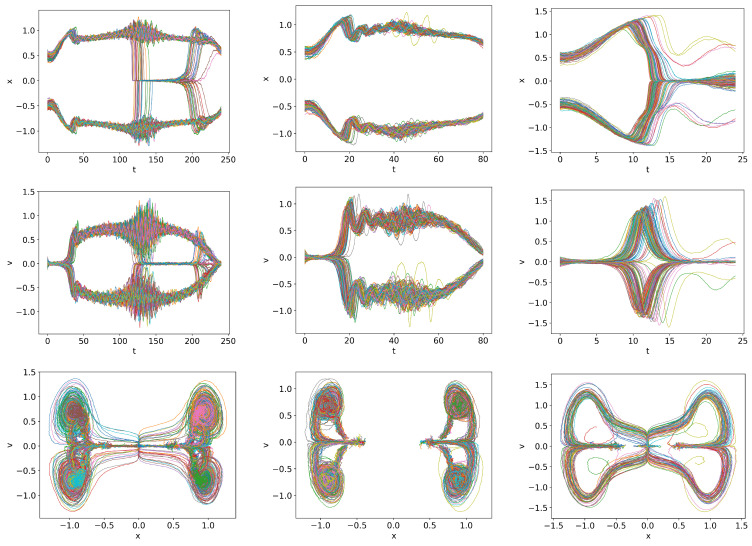
Initial condition $p\left(x,v,0\right)\propto {\exp[-((|x|-0.5)}^{2}+v^{2})/5\cdot 10^{-3}]$ and $D_{x}=D_{v}=10^{-4}$ . $Q_{c}=0.832$ . $Q=0.01t,t_{s}=120$ , $Q=0.03t,t_{s}=40$ and $Q=0.1t,t_{s}=12$ in first, second and third columns respectively.

First, let us look at the detailed dynamics for $Q=0.01,t_{s}=120$ in Figure 11. For $t>t_{s}$ , some of the *x* and *v* trajectories stay in the dithering state, while some others are attracted to the H-mode, followed by their eventual convergence to a dithering state. This is less pronounced for the faster ramping $Q=0.03t,t_{s}=40$ and $Q=0.1t,t_{s}=12$ due to the time-lag between the instantaneous Q(t) and the system’s response and consequently, due to the prevalence of dithering at the start of the ramp-down at $t=t_{s}$ .

**Figure 12:**
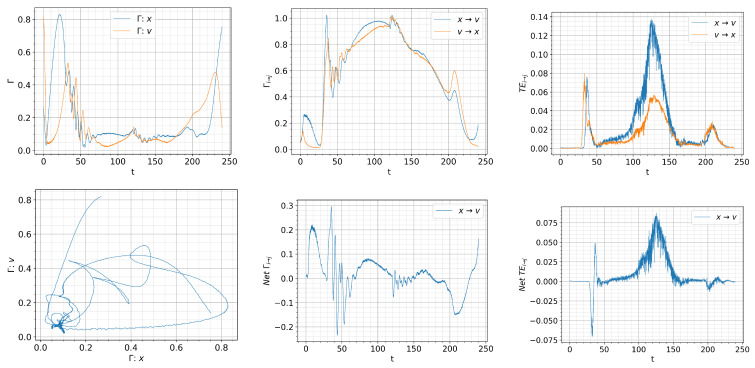
Initial condition $p\left(x,v,0\right)\propto {\exp[-((|x|-0.5)}^{2}+v^{2})/5\cdot 10^{-3}]$ and $D_{x}=D_{v}=10^{-4}$ . $Q_{c}=0.832$ . $Q=0.01t,t_{s}=120$ . Top: from left to right, information rate, causal information rate and transfer entropy; bottom: from left to right, phase portrait, net causal information rate and net transfer entropy.

**Figure 13:**
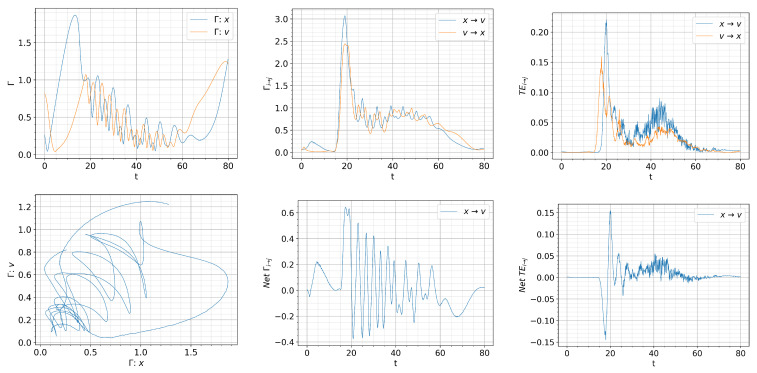
Initial condition $p\left(x,v,0\right)\propto {\exp[-((|x|-0.5)}^{2}+v^{2})/5\cdot 10^{-3}]$ and $D_{x}=D_{v}=10^{-4}$ . $Q_{c}=0.832$ . $Q=0.03t,t_{s}=40$ . Top: from left to right, information rate, causal information rate and transfer entropy; bottom: from left to right, phase portrait, net causal information rate and net transfer entropy.

**Figure 14:**
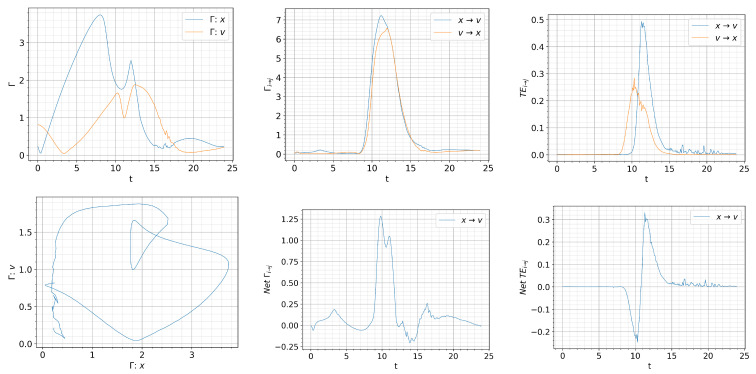
Initial condition $p\left(x,v,0\right)\propto {\exp[-((|x|-0.5)}^{2}+v^{2})/5\cdot 10^{-3}]$ and $D_{x}=D_{v}=10^{-4}$ . $Q_{c}=0.832$ . $Q=0.1t,t_{s}=12$ . Top: from left to right, information rate, causal information rate and transfer entropy; bottom: from left to right, phase portrait, net causal information rate and net transfer entropy.

Finally, in comparison with Figure 8, Figure 9 and Figure 10, Figure 12, Figure 13 and Figure 14 exhibit more robust oscillations in the information rates and causal information rates, but not much in transfer entropy. Of particular note is that the back transition from the H-mode to the dithering state around t≈150−180 is well captured by the net positive causal information rate $\Gamma_{x\to v}-\Gamma_{v\to x}>0$ , followed by the suppression of turbulence by zonal flows with the net negative causal information rate $\Gamma_{x\to v}-\Gamma_{v\to x}<0$ for 180<t<230 approximately. In comparison, such directional behaviour is not seen in the transfer entropy. Also, a similar conclusion on the utility of the causal information rate can be drawn for the faster ramping up/down in Figure 13 and Figure 14.

## 7. Comparison between Additive and Multiplicative Noise for a Mirror-Symmetric Q(t)

**Figure 15:**
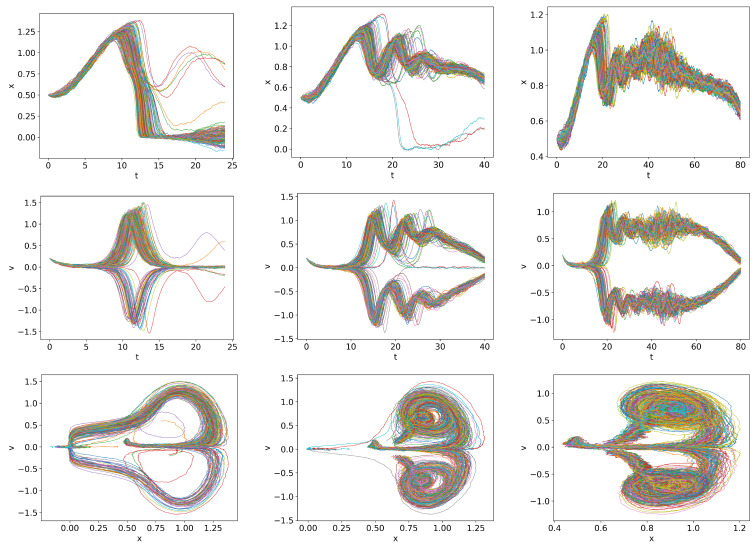
Additive zonal noise. From left to right, $Q=0.1t,t_{s}=12$ , $Q=0.05t,t_{s}=20$ and $Q=0.03t,t_{s}=40$ . Delta initial condition x(0)=0.5 and v(0)=0.2 , $Q_{c}=0.950$ .

**Figure 16:**
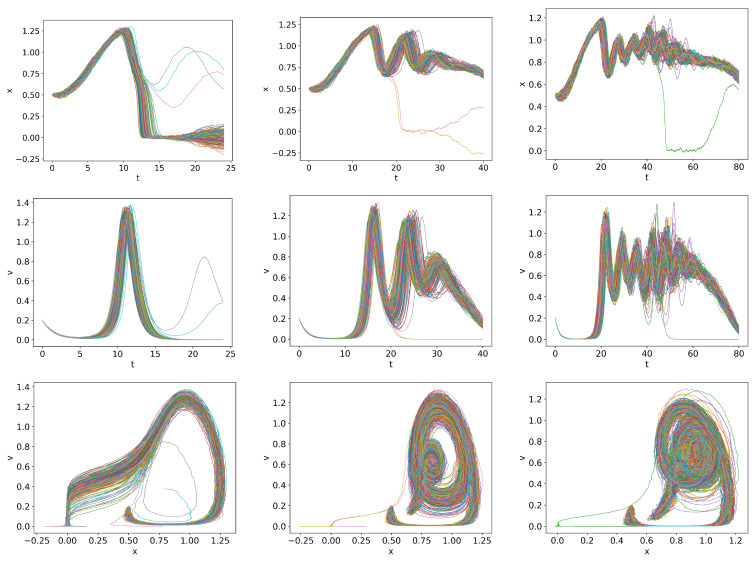
Multiplicative zonal noise. From left to right, $Q=0.1t,t_{s}=12$ , $Q=0.05t,t_{s}=20$ and $Q=0.03t,t_{s}=40$ . Delta initial condition x(0)=0.5 and v(0)=0.2 , $Q_{c}=0.950$ .

There has been a suggestion of zonal flow noises that are generated internally, e.g., through an incoherent turbulence interaction [53]. It is thus of interest to investigate the possibility where zonal flows arise from internal stochastic noise. To this end, we consider multiplicative zonal noise by replacing η in Equation (17) with ηv . Effectively, ηv represents a stochastic growth rate of zonal flow. This will also allow us to check the robustness of our results discussed above. We use an initial distribution centred around x(0)=0.5 and v(0)=0.2 and the strength $D_{x}=D_{v}=10^{-4}$ of the stochastic noises ξ and η . To highlight the key difference between the additive and multiplicative zonal noises, we consider the forward and backward processes associated with the L-H and H-L transitions using a mirror-symmetric *Q* around time $t=t_{s}$ when *Q* takes its maximum value $Q_{max}=1.2$ ; for $t<t_{s}$ , *Q* linearly increases, while for $t>t_{s}$ , it linearly decreases. The three different ramping scenarios Q=0.1t,0.05t,0.03t are used. The results for the additive and multiplicative noises are shown in Figure 15 and Figure 16, respectively, where the first, second, and third columns correspond to $Q=0.1t,t_{s}=12$ , $Q=0.05t,t_{s}=20$ , and $Q=0.03t,t_{s}=40$ , respectively.

Comparing Figure 15 and Figure 16, we observe that the overall evolution of *x* is quite similar but the H-mode trajectories appear in the multiplicative case in Figure 16 when the power takes its maximum value ( Q≈1.3) . Also, a more robust H-mode characteristic persists for a longer time in the backward process for the multiplicative zonal noise, increasing hysteresis. Mathematically, it is because a multiplicative *v* noise has the property of generating smaller values of *v* towards v=0 . This can indeed be seen from the trajectories of *v* in the third column, where v=0 persists over a long time interval 40<t<80 . Another difference between the additive and multiplicative noise cases is that stochastic trajectories with v<0 are not generated from the initial condition v(0)>0 , as *v* maintains its zero value once it becomes zero because of ηv=0 for v=0 .

## 8. Conclusions

In this paper, we presented the stochastic dynamics of the 2D prey–predator L-H transition model with GPU-based simulations. We proposed an instantaneous transfer entropy to deal with time-varying statistics and compared it with the causal information rate. The main conclusions from our investigations are as follows.

- Stochastic noise induces different trajectories that are attracted to the H-mode and dithering states, undergoing the L-H transition at different times.
- Stochastic noise induces the appearance of the H-mode at a lower input power while making the complete transition to the H-mode at a larger input power than expected from the deterministic model.
- Stochastic noise can induce stochastic switching between the two competing attractors (H-mode and dithering), leading to alternations between the two with intermittent bursts.
- Stochastic noise induces an uncertainty in the input power and a more gradual transition.
- A rapid temporal change in the input power increases the uncertainty in the input power.
- The power threshold appears to depend on how the strong turbulence and zonal flows are in the L-mode.
- Self-regulation between turbulence and zonal flows is well-characterized by the competition between and the matching of their information rates.
- The causal relation is captured by the causal information rate much better than the instantaneous transfer entropy.
- Internal stochastic zonal noise has similar effects to additive zonal noise and tends to increase hysteresis.

Our results reveal different contributing factors to the uncertainty (scatters) in the power threshold, including the initial conditions $x_{0},v_{0}$ , stochastic noise, and power ramping rate. This is an interesting result in view of the large variations in power threshold observed experimentally [23,54]. In particular, [23] applied a comprehensive statistical analysis of a large experimental dataset to establish the relation between threshold power and several machine parameters, e.g., finding that the power threshold can vary up to a factor of five at a fixed plasma density (e.g., see Figure 5 in [23]). This large variation in power threshold can arise from different L-mode turbulence characteristics, which would correspond to different initial conditions $x_{0},v_{0}$ in our study, from stochasticity due to mini-avalanches, as secondary effects of hidden variables (e.g., magnetic configurations, divertor geometry, neutral density, etc. that do not appear in the power threshold scaling relations given by plasma density, toroidal magnetic fields, surface area, etc.), or from different power ramping scenarios.

As noted in Section 4, the initial conditions mimicking strong turbulence in the L-mode ( $x_{0}\gg 0.9$ ) generate strong zonal flows, damping turbulence in turn and leading to a lower $Q_{c}$ , while a strong initial zonal flow ( $v_{0}\gg 0.7$ ) rapidly damps turbulence, again leading to a lower $Q_{c}$ . This suggests that larger $x_{0},v_{0}$ can represent any physical mechanism that can facilitate a quick damping of turbulence, e.g., through zonal flows. One example would be small collisionality, which was shown to decrease the size of coherent structure (shear flows) and turbulence level [55] in edge plasmas. In particular, small collisionality is attained for a low plasma density in the high-density branch (see, e.g., [56]) where the power threshold decreases with decreasing plasma density (see Figure 3 of [23]). Thus, our results of $Q_{c}$ decreasing with larger $x_{0},v_{0}$ in the region $x_{0}>0.9,v_{0}>0.7$ would correspond to a decreasing $Q_{c}$ with decreasing plasma density (with a lower collisionality) in the high-density branch. This warrants further study, which will require a synergistic analysis of characterizing the L-mode turbulence statistical property for different machine parameters, including hidden variables when measuring power thresholds experimentally. Furthermore, our result of the largest $Q_{c}$ for persistent dithering before the transition to the H-mode for $x_{0}∼0.9,v_{0}∼0.7$ suggests that the dithering-to-H-mode transition may require a higher power threshold than a sharp H-mode transition, which needs to be explored further in the future.

It remains for the future work to analyze the temporal and spatial dynamics involved in the L-H transition, such as profile steepnesses and shear flow poloidal widths, which could not be addressed in our reduced model. Nevertheless, our reduced model has the merit of allowing us to perform a thorough exploration of different scenarios and learn new lessons that were inaccessible from the previous deterministic, stationary, or mean-field time approaches. It will also be of interest to extend the work to perform statistical analysis of the edge localized modes (ELMs), e.g., by using stochastic simulations to extend our previous work (e.g., [57]).

## Data Availability

Data are available from the authors. The data are not publicly available due to large amounts of raw numerical output are simply not useful.

## Appendix A. Derivation of the Equality of Equations (6) and (7)

To obtain Equation (6) from Equation (7), we use the Taylor expansion

(A1)
$$\begin{array}{ccc} & & p(X_{j},t+dt;X_{i},t)\\ & & =\lim_{t_{1}\to t^{+}}\left[p\left(X_{j},X_{i},t\right)+dt\partial_{t_{1}}p\left(X_{j},t_{1};X_{i},t\right)+\frac{{\left(dt\right)}^{2}}{2}\partial_{t_{1}t_{1}}p\left(X_{j},t_{1};X_{i},t\right)\right]\\ & & \; \end{array}$$

for small dt to leading order $O({\left(dt\right)}^{2})$ to expand the logarithm term in Equation (7) as follows:

(A2)
$$\begin{array}{ccc} & & \ln\left[\frac{p(X_{j},t+dt;X_{i},t)}{p(X_{j},X_{i},t)}\right]\\ & & =\lim_{t_{1}\to t^{+}}\left[dt\frac{\partial_{t_{1}}p\left(X_{j},t_{1};X_{i},t\right)}{p(X_{j},X_{i},t)}+\frac{{\left(dt\right)}^{2}}{2}\frac{\partial_{t_{1}t_{1}}p\left(X_{j},t_{1};X_{i},t\right)}{p(X_{j},X_{i},t)}-\frac{{\left(dt\right)}^{2}}{2}{\left(\frac{\partial_{t_{1}}p\left(X_{j},t_{1};X_{i},t\right)}{p(X_{j},X_{i},t)}\right)}^{2}\right]\\ & & \; \end{array}$$

to leading order $O({\left(dt\right)}^{2})$ . Here, we used $\ln\left(1+x\right)\approx x-x^{2}/2+O\left(x^{3}\right)$ for small *x*. We plug Equation (A2) into Equation (7), use Equation (A1) once more, and then simplify terms using the total probability conservation $\int dX_{i}dX_{j}\;p\left(X_{i},X_{j},t\right)=1$ (that is, $\int dX_{i}dX_{j}\;\partial_{t_{1}}p\left(X_{i},t_{1};X_{j},t\right)=\int dX_{i}dX_{j}\;\partial_{t_{1}t_{1}}p\left(X_{i},t_{1};X_{j},t\right)=0$ ) to obtain Equation (6).

## Appendix B. Additional Results

Figure A1 provides the trajectories of x,v for the initial conditions x(t=0)=0.5 and v(t=0)=0.01 , which are slightly different from those in Figure 2.
